# TRAF1 Signaling in Human Health and Disease

**DOI:** 10.3389/fimmu.2018.02969

**Published:** 2018-12-18

**Authors:** Maria I. Edilova, Ali A. Abdul-Sater, Tania H. Watts

**Affiliations:** ^1^Department of Immunology, University of Toronto, Toronto, ON, Canada; ^2^School of Kinesiology and Health Science, York University, Toronto, ON, Canada

**Keywords:** TNFR superfamily, signaling, toll-like receptor, linear ubiquitination, cancer, autoimmunity, chronic viral infection

## Abstract

Tumor necrosis factor receptor (TNFR) associated factor 1 (TRAF1) is a signaling adaptor first identified as part of the TNFR2 signaling complex. TRAF1 plays a key role in pro-survival signaling downstream of TNFR superfamily members such as TNFR2, LMP1, 4-1BB, and CD40. Recent studies have uncovered another role for TRAF1, independent of its role in TNFR superfamily signaling, in negatively regulating Toll-like receptor and Nod-like receptor signaling, through sequestering the linear ubiquitin assembly complex, LUBAC. TRAF1 has diverse roles in human disease. TRAF1 is overexpressed in many B cell related cancers and single nucleotide polymorphisms (SNPs) in TRAF1 have been linked to non-Hodgkin's lymphoma. Genome wide association studies have identified an association between SNPs in the 5′ untranslated region of the TRAF1 gene with increased incidence and severity of rheumatoid arthritis and other rheumatic diseases. The loss of TRAF1 from chronically stimulated CD8 T cells results in desensitization of the 4-1BB signaling pathway, thereby contributing to T cell exhaustion during chronic infection. These apparently opposing roles of TRAF1 as both a positive and negative regulator of immune signaling have led to some confusion in the literature. Here we review the role of TRAF1 as a positive and negative regulator in different signaling pathways. Then we discuss the role of TRAF1 in human disease, attempting to reconcile seemingly contradictory roles based on current knowledge of TRAF1 signaling and biology. We also discuss avenues for future research to further clarify the impact of TRAF1 in human disease.

## Introduction

Tumor necrosis factor receptor (TNFR)-associated factors (TRAF) proteins play important roles in the immune system as key intracellular signaling molecules in TNFR, Toll-like receptor (TLR), cytokine, and antigen receptor signaling pathways (1). While TRAF2 is constitutively expressed and its transcript can be found in almost all tissues, TRAF1 is an NF-κB inducible protein, and under normal conditions has more limited expression in the spleen, lung, and testis (2). Evidence for TRAF1 as both a positive and negative regulator of immune signaling has led to some confusion in the literature. Here we first discuss the role of TRAF1 in TNFR and TLR signaling pathways and then discuss what is known about the impact of TRAF1 in human disease, with references to its specific roles in different pathways, attempting to reconcile these seemingly contradictory roles. Finally, we discuss the outstanding questions in the field and implications for therapy.

## Role of TRAF1 in TNFR Signaling

**Figure 1:**
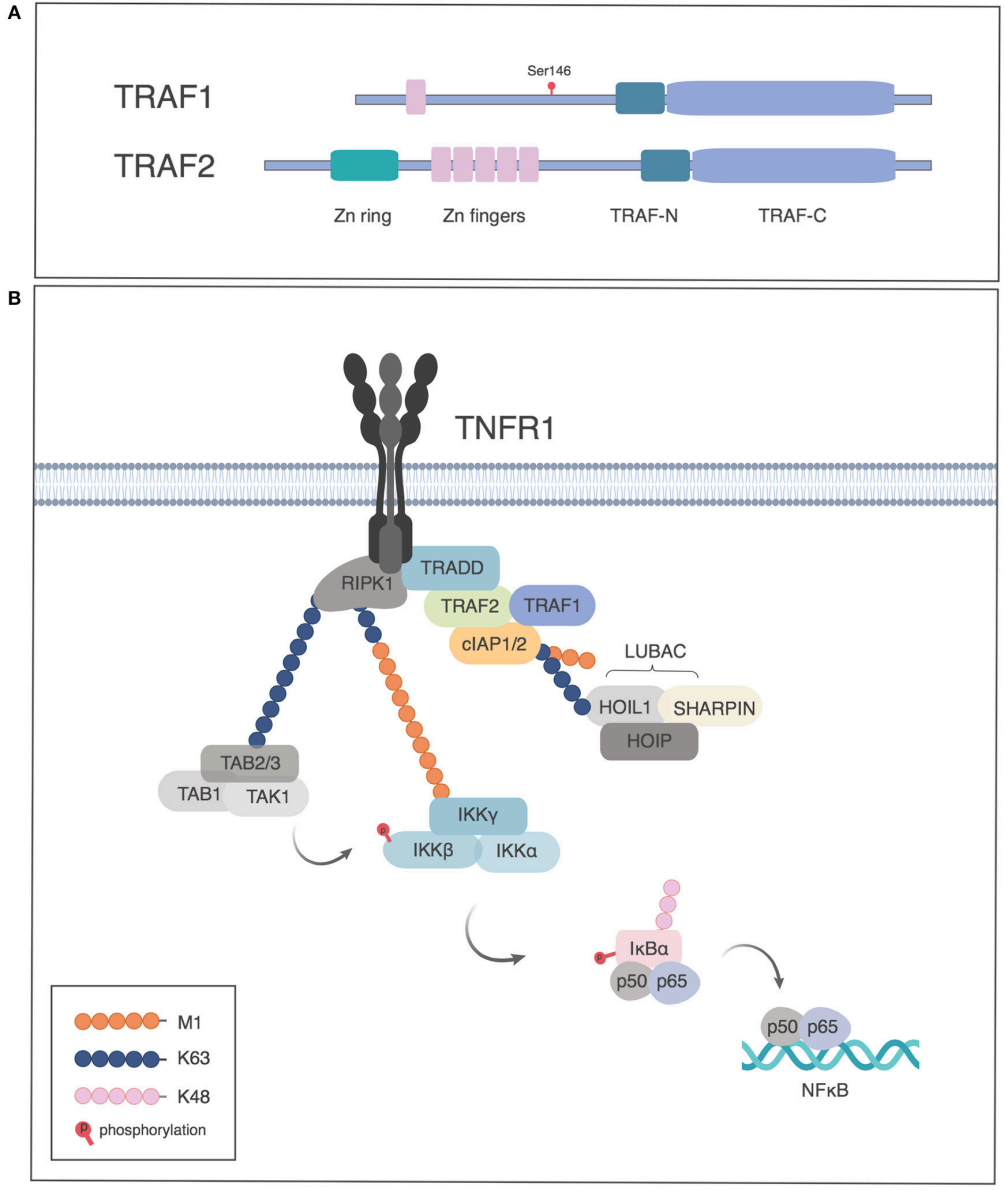
TRAF1 and TRAF 2 proteins in TNFRI signaling. **(A)** Schematic of TRAF1 and 2 structure, indicating the site of phosphorylation of human TRAF1 by PKN1. **(B)** Role of TRAF1 and 2 in activation of NF-κB by TNFRI. TNFRI recruits TNFR associated death domain, TRADD, which in turn recruits TRAF2. TRAF1 associates with the TRADD/TRAF2 complex and the TRAF complex recruits the cellular inhibitors of apoptosis protein (cIAP1 or 2), which have E3 ligase activity to add K63-Ub to RIP1, which leads to recruitment of the TAB-TAK1 complex. The linear ubiquitin assembly complex (LUBAC) is recruited by K63-Ub and adds M1-Ub chains, which in turn recruit the IKK complex through binding NEMO. The TAB/TAK1 complex activates IKK which in turn phosphorylates IκB, leading to proteasome dependent degradation of IκB and release of p50/p65 to the nucleus. Figures generated in Biorender, adapted from Wertz et al. (3).

TRAF1 was originally identified along with TRAF2 in immunoprecipitates of TNFR2 (2). TRAF2 is the prototypical TRAF protein and contains a RING finger domain, a series of Zinc fingers followed by the conserved TRAF domain. The TRAF domain, conserved among TRAFs 1 through 6, consists of the TRAF-N, a coiled coil region responsible for homo-, or hetero-oligomerization of TRAF proteins and a C-terminal domain, TRAF-C, also referred to as the meprin and TRAF homology (MATH) domain, which is responsible for TRAF recruitment to the cytoplasmic tails of TNFRs. TRAF1 differs from TRAF2 in lacking the N-terminal RING finger required for NF-κB activation and in having only one Zinc finger (Figure 1A) and as such resembles a dominant negative form of TRAF2 (4). The crystal structure of the TRAF1 TRAF domain shows that like other members of the TRAF family, the TRAF C domain forms a mushroom shaped cap and the TRAF N domain forms a stalk with a coiled coil structure, albeit with some specific differences from other TRAFs in the location of several loops in the TRAF domain and the position of the coiled coil α helices (5, 6). The structure of TRAF1 has also been solved in a complex with the protein TANK, TRAF family member-associated NF-kappa B activator (7).

Under normal conditions, TRAF1 expression is largely limited to activated immune cells, including myeloid and lymphoid cells. TRAF1 is present at minimal levels in resting lymphocytes and monocytes and its expression is increased upon activation through the NF-κB pathway (8). TRAF1, along with TRAF2 and the cellular inhibitors of apoptosis (cIAP1 and cIAP2), is required to suppress TNF-induced apoptosis in NF-κB-deficient cell lines (9). *Traf1*^−/−^ mice are viable and fertile and have normal numbers of lymphocytes (10). However, TRAF1-deficient activated and memory T cells have impaired survival (11–13). Conversely, transgenic expression of TRAF1 in mice reduces antigen induced cell death in T cells (14). These data are consistent with a prosurvival role for TRAF1 in lymphocytes.

### Role of TRAF1 in the Classical NF-κB Pathway Downstream of TNFRs

Pro-survival members of the TNFR superfamily activate NF-κB and mitogen activated protein kinase (MAPK) pathways. For TNFRI, TRAF proteins are recruited indirectly through TRADD (Figure 1B), whereas TNFRs that lack death domains recruit TRAFs directly (1). The TRAF proteins, in turn, recruit cIAP1 or 2. The NF-κB pathway involves three kinds of ubiquitination. cIAPs have E3 ligase activity to add K63-linked polyubiquitin (K63-Ub) to receptor-interacting serine/threonine-protein kinase 1 (RIPK1). The K63-Ub provides a substrate for addition of linear ubiquitin as well as for recruitment of TGFβ-associated kinase (TAK1) and TAK binding protein (TAB), required for Inhibitor of kappa B kinase (IKK) and MAPK activation (15, 16). Linear ubiquitination involves the addition of polyubiquitin polymerized through the M1 position (M1-Ub) (17–19). The addition of M1-Ub is catalyzed by the linear ubiquitin assembly complex (LUBAC), which consists of three subunits: HOIL, HOIP, and SHARPIN. LUBAC is recruited by K63-Ub and can also modify K63-Ub to make hybrid molecules (19). These ubiquitin modifications serve as scaffolds whereby linear ubiquitin recruits the IKK complex, consisting of IKKα, IKKβ, and Nemo/IKKγ, which itself can also be modified by M1- and K63-Ub. K63-Ub recruits TAK/TAB, leading to activation of the IKK complex. M1-Ub is important in NF-κB activation downstream of TNFR as well as TLRs and NLRs (18–22). Activated IKK in turn phosphorylates the Inhibitor of κB (IκB), leading to its K48-Ub modification and degradation, allowing NF-κB translocation into the nucleus (Figure 1B) (16).

TRAF1 enhances survival signaling downstream of a subset of TNFR family members, including TNFR1, TNFR2, CD40, 4-1BB (CD137), and the EBV-encoded TNFR family member latent membrane protein (LMP)-1, by enhancing classical NF-κB and MAPK activation (12, 23–29). The coiled coil domain of TRAF1 when mixed with that of TRAF2 spontaneously forms a 1:2 heterotrimer and this complex asymmetrically recruits the BIR domain of cIAP2 (30). The (TRAF2)_2_TRAF1 coiled coil heterotrimer, is more efficient in recruitment of cellular cIAPs than the TRAF2 homotrimer, and thus TRAF1 provides an NF-κB induced positive feedback loop to enhance TRAF2-dependent signaling (30). *Traf1*^−/−^ dendritic cells (DC) show increased apoptosis and marked deficiency in classical NF-κB activation after CD40 stimulation, implicating TRAF1 in sustaining TRAF2-dependent signaling through CD40 (26). Similarly, in B cells, TRAF1 and TRAF2 were found to cooperate in induction of NF-κB and JNK activation (25). The absence of TRAF1 in T cells leads to impaired NF-κB and ERK activation downstream of 4-1BB and accumulation of the pro-apoptotic molecule BIM (11–13, 28).

### TRAF1 Prevention of TRAF2 Degradation

Beyond its demonstrated role in collaboration with TRAF2 in recruiting cIAPs, TRAF1 can prevent proteasome-dependent degradation of TRAF2 downstream of CD40, 4-1BB and TNFR2 signaling, thereby sustaining TRAF2 dependent signaling (12, 23, 25, 26). cIAPs not only add K63-Ub to RIP, but also have E3 ligase activity for adding K48-Ub which can lead to TRAF2 degradation (31). TRAF1 can prevent this effect of cIAPs during TNFR family signaling, although the mechanism of this protection remains to be elucidated. How much of the role of TRAF1 is due to improved recruitment of cIAP by the TRAF1/2 heterotrimer over the TRAF2 homotrimer (30) and how much is due to TRAF1 preventing TRAF2 degradation, or whether the two are interrelated, is unclear.

### Post-translational Modifications of TRAF1

**Figure 2:**
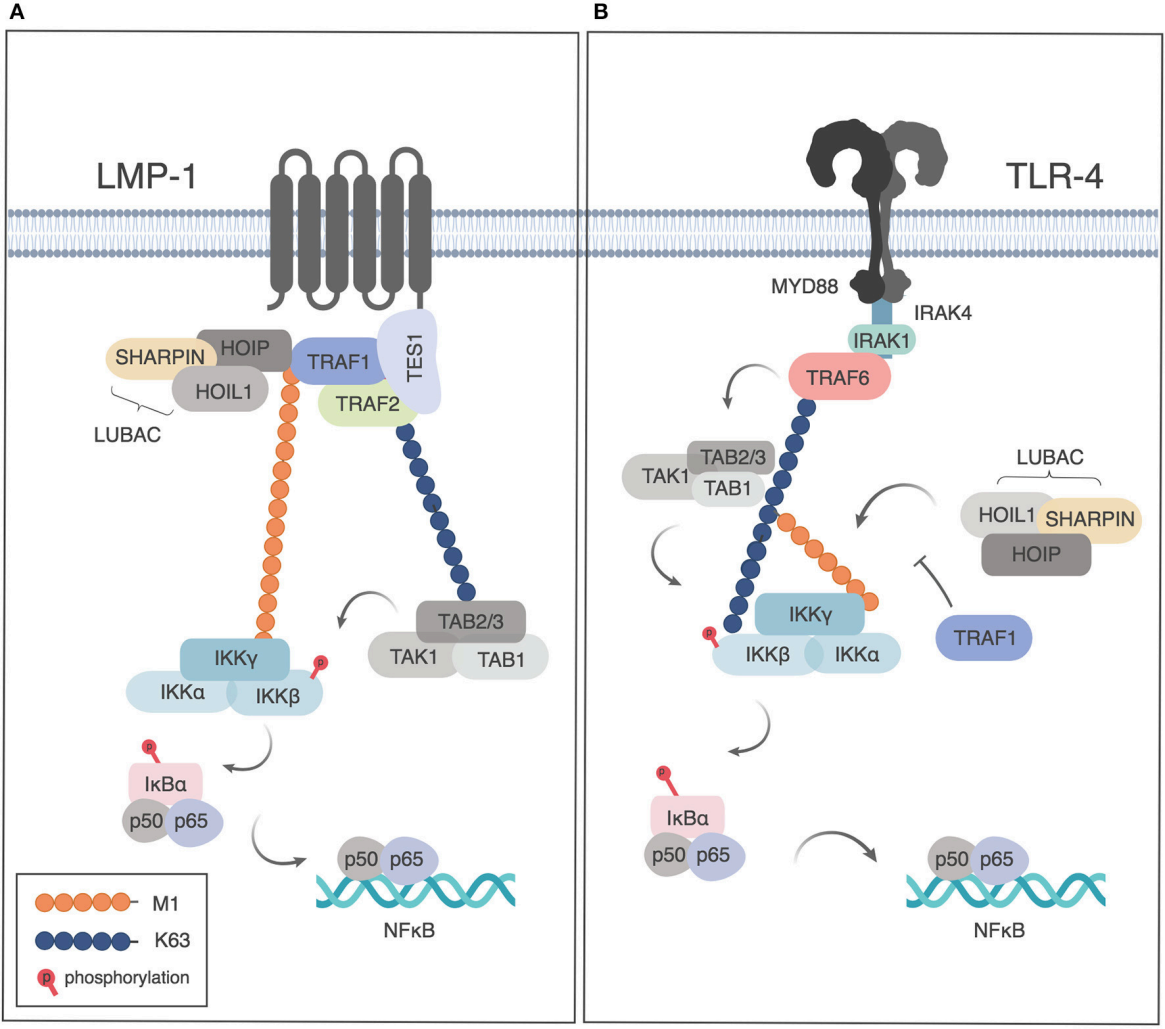
Role of TRAF1 and linear ubiquitination downstream of LMP1 and TLR4. **(A)** TRAF1 is recruited to the TES domain of LMP1 and modified by linear ubiquitination downstream of LMP1 signaling, leading the recruitment of NEMO and NF-κB activation. Adapted from Greenfeld et al. (29) **(B)** Downstream of TLR signaling, TRAF1 sequesters LUBAC, thereby limiting linear ubiquitination of NEMO and restricting NF-κB activation. Figures generated in Biorender, adapted from Abdul-Sater et al. (32).

TRAF1 and LUBAC can be co-immunoprecipitated with the TES domain of the EBV-encoded TNFR family member LMP1, when TRAF1, and the TES construct are overexpressed in HEK293 cells (29). LMP1-dependent signaling results in M1-Ub modification of TRAF1. TRAF2, but not cIAP1 or 2, were found to be important in LUBAC recruitment in this model (29). As TRAF1 was found to increase NF-κB signaling downstream of LMP1, the authors proposed that this M1-Ub modification of TRAF1 was important in IKK recruitment and showed that LMP1 and TRAF1 could co-localize with an M1-Ub sensor that contained the ubiquitin binding domains of ABIN1 and NEMO/IKK-γ (Figure 2A). Consistently, knockdown of TRAF1 or HOIP resulted in reduced proliferation of a large cell lymphoma cell line (29).

The protein kinase C related kinase, PKN1 binds to and phosphorylates TRAF1 on serine 146 in human and serine 139 in mouse, but does not phosphorylate TRAF2, 3 or 5 (33), despite binding to TRAF2 (34). Knockdown of PKN1 enhanced basal IKK activation in HeLa cells. In overexpression systems with FLAG-Tagged TRAF1, serine 139 of TRAF1 was found to enhance its recruitment to TNFR2 relative to an alanine mutant, and cells expressing TRAF1 S139A showed enhanced recruitment of TRAF2 to TNFR2 in 293T cells, leading to the suggestion that phosphorylation of TRAF1 allows it to compete with TRAF2 for recruitment to TNFR2 and thereby inhibits NF-κB activation (33). However, the role of PKN1 on TRAF1 biology has yet to be tested in a more physiological setting.

### Role of TRAF1 in the Alternate NF-κB Pathway

TRAF1 has also been implicated in regulation of the alternate NF-κB pathway in lymphocytes. The alternate NF-κB pathway involves degradation of p100 protein by NF-κB-inducing kinase (NIK) to the active p52 form. In resting cells, NIK is constitutively degraded due to its ubiquitination by cIAP1 and/or 2. TRAF2 and TRAF3 play non-redundant roles in this process, with TRAF2 bringing in cIAPs to the complex, and TRAF3 bringing in NIK, thereby inducing NIK degradation and preventing constitutive NF-κB activation. Accordingly, mice lacking TRAF2 or 3 have constitutive non-canonical NF-κB signaling and die of lethal inflammation (35, 36). Mice lacking TRAF1 lack this lethal inflammation (10), however T cells lacking TRAF1 are hyper-responsive to anti-CD3 (10). The hyper-responsiveness of *Traf1*^−/−^ T cells to anti-CD3 was later shown to be dependent on NIK and was associated with excessive cytokine production (28). In T cells lacking TRAF1, p100 is processed to p52 in response to anti-CD3 alone, whereas in WT cells p100 processing requires both a TCR signal and a TNFR family signal (28). The role of TRAF1 in limiting non-canonical signaling in anti-CD3 activated T cells may be due its role in preventing TRAF2 degradation or due to its role in enhancing cIAP recruitment. As TRAF1 has only limited expression, it cannot have an essential role in restraining NIK in all cell types, but primarily plays this role in the context of activated lymphocytes. As TCR signaling induces increased expression of p100, it is possible that increased regulation of NIK is required to prevent spontaneous non-canonical NF-κB induction until a costimulatory signal is received (28).

TNFR family members induce activation of the alternate NF-κB pathway by inducing degradation of TRAF2 or TRAF3, usually with delayed kinetics compared to the activation of the classical NF-κB pathway (28, 35, 36). TRAF1 can positively regulate this process (37), likely through recruitment of cIAPs, which are also involved in degrading TRAF3 leading to alternative NF-κB activation (28, 35, 36). Another study, which used overexpression of both TRAF1 and NIK, showed that TRAF1 could bind to NIK and thereby prevent NIK degradation in A549 cells (38); however, the relevance of this interaction in a physiological setting is not clear. Thus, the role of TRAF1 in the alternative NF-κB pathway depends on whether there is active TNFR signaling going on. How TRAF1 promotes TRAF3 degradation to induce the alternative NF-κB activation or prevents cIAP-mediated TRAF2 degradation to allow classical NF-κB activation remains to be elucidated.

### Does TRAF1 Also Play a Negative Role in TNFR Signaling?

Overexpression of TRAF1 in cell lines can lead to inhibition of TRAF2-mediated NF-κB activation (39). Additionally, a caspase-induced cleavage product of TRAF1 can interfere with TRAF2-mediated survival signaling (40, 41). This is thought to be due to competition for binding to TRAF binding sites, thereby preventing TRAF2 recruitment. However, as discussed above, transgenic expression of TRAF1 in lymphocytes has a pro-survival effect and loss of TRAF1 impairs T cell survival (11, 14). Thus, in more physiological systems with normal lymphocytes TRAF1 plays a largely positive role in NF-κB signaling. Nonetheless, analysis of TRAF1- deficient mice showed that anti-CD3 stimulated *Traf1*^−/−^ cells hyper-proliferated in response to anti-CD3 alone or in response to TNF and the response of the activated T cells to TNF was specifically blocked by antibodies to TNFR2, leading to the suggestions that TRAF1 is a negative regulator of TNF signaling (10). As discussed above, *Traf1*^−/−^ T cells hyperproliferate due to increased activation of the alternative NF-κB pathway, and these effects might have confounded the interpretation of anti-CD3 activated T cells responding to TNF. In the same study *Traf1*^−/−^ mice were found to have increased TNF-induced skin necrosis. However, as will be discussed below, *Traf1*^−/−^ mice have enhanced responses to TLR signaling, and it is possible that the damage to the skin caused by TNF allowed enhanced TLR-signaling due to skin associated microbes, and thus the negative regulatory role observed might have reflected signals through TLRs, rather than TNFRs. Other studies have clearly shown a role for TRAF1 in enhancing NF-κB signaling downstream of TNFRs (23). Thus, the weight of the evidence suggests that under conditions of physiological expression in viable lymphocytes, TRAF1 plays a largely positive role in NF-κB induction and lymphocyte survival downstream of TNFRs. On the other hand, if caspases are activated, it is possible that the caspase-induced cleavage product of TRAF1 contributes to cell death.

## TRAF1 as a Negative Regulator of TLR and NLR Signaling

Two studies have demonstrated negative regulation of NF-κB signaling by TRAF1 downstream of TLRs or NLRs, albeit by different mechanisms. Abdul-Sater et al. showed that TRAF1 binds directly to all three components of LUBAC (SHARPIN, HOIP and HOIL), thereby preventing linear ubiquitination of NEMO, and thus limiting downstream NF-κB activation after TLR or NLR signaling (32) (Figure 2B). TRAF1 binding to LUBAC components was dependent on the presence of the MATH domain of TRAF1 and independent of TRAF2 or TNF signaling. TRAF1 binding was largely abrogated by deletion of the HOIP or HOIL NZF domain, a conserved domain required for NEMO recruitment that is found in all 3 LUBAC components (32). As the interaction of TRAF1 with LUBAC components was shown using purified proteins, this demonstrates a role for TRAF1 independently of TRAF2, and thus distinct from its role in TNFR signaling pathways. Of interest, the interaction of TRAF1 with HOIP and HOIL had also been suggested but not further analyzed in a study of protein-protein interactions downstream of microbial stimulation leading to interferon induction (42).

In another study, TRAF1 was identified in a yeast 2-hybrid screen that used the TLR signaling molecule TIR-domain-containing adapter-inducing interferon-β (TRIF) as bait. The TRAF-C domain of TRAF1 was found to bind to the TIR domain of TRIF. TRAF1 overexpression blocked TRIF-dependent NF-κB reporter activation in 293 cells, dependent on the caspase-sensitive cleavage site in TRAF1 (43). However, the physiological role of this cleaved form of TRAF1 in primary cells has not been demonstrated.

## Summary TRAF1 Signaling

In summary, TRAF1 contributes to signaling in the TNFR signaling pathway as part of a complex with TRAF2, where it can promote classical NF-κB activation through cIAP recruitment and possibly through stabilization of TRAF2. Later, TRAF1 may also contribute to induction of the alternate NF-κB pathway, again through cIAP recruitment. TRAF1 can also contribute to NF-κB activation independently of cIAPs downstream of LMP1 signaling, through becoming a substrate for linear ubiquitination, possibly contributing to recruitment of IKKγ/NEMO. Conversely, during TLR or NLR signaling, TRAF1 can sequester LUBAC to negatively regulate NF-κB activation. How these opposing roles of TRAF1 in different contexts impact human disease will be discussed in the remainder of the article.

## Role of TRAF1 in Cancer

### B Cell Cancers

There is extensive evidence for altered expression of TRAF1 in lymphoid malignancies (44–46). Many human B malignancies including B cell chronic lymphocytic leukemia cells (CLL), non-Hodgkin lymphoma (NHL), and Burkitt's lymphomas exhibit constitutive signaling via TRAF1 binding TNFRs, such as CD30 and the EBV protein LMP1, and this in turn is thought to contribute to high levels of TRAF1 expression via NF-κB signaling (24, 46, 47). Additionally, B-CLL receive signals through CD40L, and this can drive CD40-dependent TRAF1 expression (48, 49). Immunological analysis of NHL revealed TRAF1 overexpression in 48% of cases, and the same study showed the highest levels of TRAF1 protein in refractory CLL (45). Analysis of TRAF1 in Hodgkin–Reed–Sternberg cells of highly proliferating tumors such as Hodgkin lymphoma (HL) and anaplastic large cell lymphoma led to the suggestion that TRAF1 contributes to apoptosis resistance downstream of CD30, and therefore plays an important role in the pathogenesis of classical HL (50). Mediastinal large B-cell lymphoma (MLBCL), a subtype of diffuse large B-cell lymphoma (DLBCL), and HL have a shared survival pathway with high levels of expression of TRAF1 and activation of the NF-κB pathway (51). Anaplastic large-cell lymphomas carrying anaplastic lymphoma kinase (ALK) have a relatively good prognosis, however aggressive forms exist. A translocation that fused the TRAF1 and ALK genes was observed in one patient and was associated with upregulation of ALK and NF-κB pathways. Treatment of TRAF1-ALK cells with proteasome inhibitors, to block the NF-κB pathway, resulted in p50/p52 downregulation and inhibition of lymphoma growth (52–54). Other evidence for the importance of TRAF1 in lymphoma comes from the identification of single nucleotide polymorphisms (SNPs) in the region between TRAF1 and complement factor 5 (TRAF1-C5 locus) that predisposes to lymphoma, although the precise causative SNP has not been identified (55, 56).

The importance of TRAF1 in lymphoma has also been validated in mouse models. Mice that overexpress a truncated form of TRAF2 that is thought to mimic TRAF1 develop lymphadenopathy and splenomegaly due to polyclonal B cell expansion. *In vitro*, these B cells exhibit comparable proliferation rates to wild-type B cells but have markedly increased survival and resistance to apoptosis induced by dexamethasone and chemotherapeutic agents. The histopathologic features of these B cells are consistent with mouse small B cell lymphoma progressing to leukemia and exhibit many similarities to human chronic lymphocytic leukemia (57). A more direct test of the role of TRAF1 in lymphomagenesis was carried out with mice engineered to express a constitutively active NF-κB2 mutant. These mice have expanded peripheral B cell populations and develop small B cell lymphomas. The mutation has no apparent effect on the proliferation of B cells but renders them resistant to apoptosis-induced by cytokine deprivation and mitogenic stimulation. The lymphocytes and lymphoma cells from these transgenic mice express high levels of TRAF1. Importantly, crossing the NF-κB2 mutant mice with *Traf1*^−/−^ mice re-established B cell homeostasis, implicating TRAF1 in the pathogenesis of lymphoma (58).

### Other Cancers

According to the Human protein atlas (www.proteinatlas.org), TRAF1 can be found in other cancers besides lymphomas and CLL, including head and neck, melanoma, pancreatic, and thyroid cancers. In addition, as discussed below, recent evidence shows that human squamous cell carcinoma and non-small cell lung carcinomas can show overexpression of TRAF1.

#### Squamous Cell Carcinoma

In human skin, TRAF1 is expressed at higher levels, as measured by histology, in actinic keratosis as well as in squamous cell carcinoma, compared to normal skin (59, 60). Since UV exposure is thought to contribute to these conditions, Yamamoto et al. tested the role of UV in induction of TRAF1 in mice. They found that TRAF1 was induced and persisted after 3 rounds of UV irradiation. Moreover, TRAF1 was required for carcinogenesis in a UV-induced mouse skin carcinogenesis model (60).

#### Non-small Cell Lung Carcinoma

Two recent studies reported that TRAF1 is overexpressed in human non-small cell lung cancer and that TRAF1 expression level inversely correlated with patient survival (61, 62). Moreover, in a urethane-induced mouse model, loss of TRAF1 decelerated tumor invasion (61). Knocking down TRAF1 expression in human lung cancer cell lines impaired phosphorylation of the oncogene serine/threonine-protein kinase, BRAF, and affected TRAF2-mediated BRAF Lys48-linked ubiquitination (61). This led to decreased BRAF protein, reduction of downstream MEK and ERK pathway activation and inhibition of growth and differentiation, ultimately leading to death of the lung cancer cells (61). In this study, the TNFR family members involved were not identified, but the studies are consistent with a role for TRAF1 in enhancing TRAF2-mediated signaling in NSCLC. A number of mutations in the TRAF1 gene have been identified in human lung cancer and several other cancers and these are discussed elsewhere in this topic (63).

## Autoimmunity, Rheumatoid-Arthritis Associated Sepsis, and Cardiovascular Disease

Genome-wide association studies first identified SNPs in the *TRAF1-C5* locus on chromosome 9 as risk factors for rheumatoid arthritis (RA) in human patients (64–69). In a study of 400 RA patients, Panoulas et al. found that TRAF1/C5 SNP rs3761847 GG homozygote status is also associated with an increased risk of death from sepsis or malignancies but not from cardiovascular disease in patients with established RA (70). In that study, 43.5% of deaths were due to infection, with 30% due to cardiovascular disease and 26% due to malignancy (70). Another study using an inception cohort of 615 recently diagnosed RA patients did not find a link between the TRAF1/C5 SNP rs10818488 and mortality in RA patients or in a non-RA elderly cohort (71). In this RA cohort (71), the leading cause of death was cardiovascular disease with only 9% dying from infections. Thus, differences in causes of death in the different cohorts might have impacted the results. Note that the rs10818488 SNP studied in (71), is in linkage disequilibrium, r^2^ value of 0.98, with the rs3761847 SNP studied by Panoulas et al. (70). Interestingly, although the two aforementioned studies found no link between the TRAF1 SNP and cardiovascular disease, a recent study has suggested there could be a link. Hessler et al. identified a TRAF1 SNP, rs2416804 as associated with carotid intima-media thickness, a marker of early stage atherosclerosis and considered a predictor of subsequent cardiovascular events (72). rs2416804 is in linkage disequilibrium with rs3761847, *r*^2^ = 0.96. Additional studies with larger cohorts representing more diverse disease outcomes will likely be required to resolve these apparent differences in TRAF1-associated disease outcomes.

SNPs in *TRAF1/C5* have also been implicated in other inflammatory and autoimmune conditions, including autoimmune thyroid diseases, juvenile idiopathic arthritis, and systemic lupus erythematosus (73–78). As several TRAF1 SNPs are in complete or almost complete linkage disequilibrium, the exact causative SNP is not known. Thus, it is not clear if the SNP that affects NHL (55), discussed in the previous section, is the same as the SNPs that affects rheumatic disease.

Increased serum levels of TRAF1 correlate with disease activity and autoantibodies in RA patients. Moreover, SNPs in the *TRAF1-C5* locus may predict the clinical response to anti-TNF therapy (79, 80). The expression of TRAF1 is also significantly higher in inflamed and non-inflamed tissues of patients with inflammatory bowel disease compared to those in control patients (81). However, as TRAF1 is an NF-κB induced gene, the finding of increased TRAF1 in patients with the SNP may relate to the increased inflammatory activity in the patients and not to the direct effect of the SNP. Therefore, to address the role of the TRAF1 SNP in human disease, our group studied healthy donors with the disease associated or disease resistant SNP, rs3761847. It was important to use healthy donors for this study, in order to assess the effect of the SNP on TRAF1 protein levels, independently of chronic inflammation (32).

Surprisingly, our group found that activated T cells as well as monocyte from healthy donors with the disease associated SNP had lower levels of TRAF1 than those with the disease resistant SNP, with an intermediate phenotype in heterozygotes. This finding was somewhat paradoxical given the positive role for TRAF1 in enhancing survival of T lymphocytes (11, 13, 14). However, around the same time our lab had found that *Traf1*^−/−^ mice had increased responses to endotoxin-induced shock (32), and as this is largely a monocyte induced disease, we decided to focus our analysis of the TRAF1 SNP on human monocytes. Consistent with a role for the TRAF1 SNP in inflammation, monocytes from healthy human subjects with the risk associated-SNP produce increased amounts of pro-inflammatory cytokines, including TNF and IL-6 in response to lipopolysaccharide (LPS). As discussed above, further investigation revealed that TRAF1 attenuated TLR-induced cytokine production by sequestering LUBAC, thereby limiting linear ubiquitination of NEMO and limiting NF-κB activation (32). Thus, donors with less TRAF1 protein have enhanced responses to TLR/NLR signaling. These findings suggest that enhanced inflammation due to innate immune signaling likely explains the enhanced disease severity in patients with the risk associated SNP. The findings also suggest that the effects of TRAF1 in limiting innate immune inflammatory signaling outweigh the effects of TRAF1 in sustaining TNFR superfamily signaling in lymphocytes. Indeed, in our study, we showed that when T cell stimulation with anti-CD3 was combined with LPS stimulation of PBMC from donors with or without the TRAF1 risk allele, the effects of TRAF1 on the TLR signaling pathway dominated (32). Over the course of a lifetime, one likely has far more exposure to short-term inflammatory stimuli than to severe infections. Signaling downstream of the TRAF1-dependent TNFR family member 4-1BB is dispensable in mild, as compared to severe influenza infection (82). Thus, the negative regulatory role of TRAF1 in limiting NF-κB during repetitive exposures to innate immune stimuli likely has a more profound effect on overall level of inflammation in humans than the detrimental effects of slightly lowered TRAF1 on TNFR superfamily-induced T cell survival, which might only become apparent during more severe infections.

## Infectious Diseases

### Human Immunodeficiency Virus

During chronic infection, the immune system must be tightly regulated to avoid pathology. These regulatory mechanisms include the persistent upregulation of inhibitory receptors such as PD1 on chronically stimulated T cells, as well as sustained production of anti-inflammatory cytokines, such as IL-10 and TGFβ (83). As many TNFRs are upregulated on activated T cells, this raised the question of how TNFR signaling is regulated during chronic infection. The TNFR family member 4-1BB is a TRAF1 binding TNFR that is normally absent from resting cells, but induced by TCR signaling, and becomes persistently upregulated on antigen-stimulated T cells during chronic infection (84). However, at the chronic phase of chronic LCMV infection, 4-1BB does not contribute to viral control, as its signaling pathway is desensitized due to TGF-β-dependent TRAF1 degradation in the chronically stimulated CD8 T cells (84). Of note, TRAF1 can be upregulated by common γ chain cytokines, including IL-7, which augments TRAF1 expression in human and mouse T cells. Moreover, treatment of mice with IL-7 prior to provision of an anti-4-1BB agonist restored responses to 4-1BB and lowered viral load (84).

Early in infection, TRAF1 is highly expressed in CD8 T cells responding to HIV, consistent with their activated phenotype. However, with progression of infection, TRAF1 levels are decreased in HIV-specific CD8 T cells in donors followed longitudinally. In a cross-sectional cohort, TRAF1 protein was higher in HIV-specific CD8 T cells from patients who were able to control HIV in the absence of drug treatment, so-called elite controllers, than in chronic progressors (84). Moreover, the frequency of TRAF1^+^ HIV-specific CD8 T cells in infected patients inversely correlated with the frequency of PD-1^hi^ exhausted T cells. The importance of TRAF1 in the CD8 T cells from elite controllers was demonstrated by siRNA-knockdown of TRAF1, which resulted in decreased ability of the CD8 T cells to eliminate HIV-infected CD4 T cells in an *ex vivo* co-culture system. Moreover, knockdown of both TRAF1 and BIM led to enhanced CD8 T cell activity compared to knockdown of TRAF1 alone, consistent with previous findings that 4-1BB can regulate BIM through TRAF1-dependent ERK activation (12, 13, 84).

IL-7 therapy has been used in clinical trials to treat HIV-infected patients whose CD4 T cell counts fail to rebound despite the successful reduction of viral load by anti-retroviral therapy (85–88). One such clinical trial offered the opportunity to monitor TRAF1 levels before or after IL-7 therapy of human subjects. Although sample size was small, there was evidence that some donors increased their level of TRAF1 in HIV-specific T cells as measured 10 weeks after the last IL-7 treatment cycle. Of interest, the level of TRAF1 in the HIV-specific T cells was strongly associated with the level of phospho-ribosomal protein S6, pS6, a downstream target of the metabolic checkpoint kinase mTOR that is associated with cell size. As TRAF1 can enhance MAPK activation downstream of TNFRs, and ERK can enhance mTOR activation through negatively regulating the negative regulator TSC2, this suggests that TRAF1 in T cells may be an important regulator of the mTOR-S6 signaling axis and may contribute to T cell fitness (89).

### Hepatitis C Virus

Hepatitis C infection of humans can result in diverse outcomes, from full resolution of infection to long-term chronic infection, which can ultimately lead to liver cirrhosis or hepatocarcinoma. Moreno-Cubero et al. recently examined Hepatitis C virus (HCV)-specific CD8^+^ T cells from patients with progressive infection and those with resolved infection (90). As with chronic HIV infection, progressive exhaustion during persistent infection with HCV was also associated with loss of TRAF1 measured directly *ex vivo* or after *in vitro* TCR stimulation. After *in vitro* T cell receptor stimulation, TRAF1 expression positively correlated with the levels of IL-7R, Mcl-1, and CD107a expression and proliferation intensity and negatively correlating with PD-1 expression. This study also confirmed the results from the HIV study that IL-7 enhanced, whereas TGF-β1 impaired TRAF1 expression in CD8 T cells from infected patients. Consistently, the serum concentration of TGF-β1 was higher in patients with persistent infection than in patients with resolved infection. Moreover, the authors showed that IL-7 plus 4-1BBL treatment *ex vivo* could improve T cell responses of chronically infected patients. In a subset of patients, characterized by slowly progressing liver fibrosis, *in vitro* treatment with anti-PD-L1, in addition to the combination of IL-7 and 4-1BBL, re-established T cell proliferation in individuals with long-lasting persistent infection, once again supporting the idea that TRAF1 is a key regulator involved in supporting specific CD8^+^ T cell responses during chronic viral infection (90).

### Epstein-Barr Virus

It has been long established that latent membrane protein-1 (LMP1) is essential for Epstein-Barr virus (EBV)-mediated lymphocyte transformation (44, 91, 92). LMP1 recruits TRAF proteins, including TRAF1 to mimic CD40 receptor signaling in EBV-infected B lymphocytes leading to the activation of NF-κB, MAPK, phosphatidylinositol 3-kinase (PI3-K), IRF7, and STAT pathways (93). TRAF1 is amongst the most highly expressed LMP1-induced target genes and is abundantly expressed in EBV-associated disorders. There is high and consistent TRAF1 overexpression in EBV-induced lymphoproliferations and Hodgkin's disease (44, 94). In addition, many cases of post-transplant lympho-proliferative disease and related disorders are TRAF1 positive (92). Siegler et al. showed that TRAF1 co-localizes with LMP1 in EBV-infected cells in tonsillar cells of infectious mononucleosis patients (95). As discussed earlier, TRAF1 associates with LUBAC and is modified by M1-Ub in the LMP1 signaling complex and this is thought to enhance IKK recruitment and NF-κB activation in EBV-infected cells (29).

## Conclusions and Future Directions

TRAF1 has diverse roles in human health and disease. TRAF1 contributes to control of chronic viral infection and can limit inflammation. This suggests that enhancing TRAF1 expression could be beneficial for both chronic infection and inflammatory diseases. Blocking TGFβ or stimulating IL-7 offer possible interventions for achieving higher TRAF1 in chronically stimulated T cells. Conversely, TRAF1 is dysregulated in cancer, where it likely contributes to a positive feedback loop that perpetuates NF-κB signaling and survival of cancers of mature B cells. TRAF1 also contributes to survival of EBV-dependent cancers through enhancing LMP1- mediated survival signaling. Interfering with TRAF1 in this process could break the cycle of NF-κB activation in these cancers. Human variations in TRAF1 correlate with increased incidence of rheumatic disease, increased mortality from sepsis in RA patients, and increased incidence of NHL. The role of TRAF1 as both a positive and negative regulator of immune responses can be attributed to its participation in diverse signaling pathways. TRAF1 is important in TNFR superfamily signaling as a complex with TRAF2 and in TLR/NLR signaling independently of TRAF2. What happens in a monocyte responding to both a TLR and a TNF signal? Does TRAF1 limit one and enhance the other simultaneously, and/or are there separate pools of TRAF1 in the cell that engage in these different functions? More work is required to understand how the diverse roles of TRAF1 play out in complex biological systems *in vivo*. TRAF1 interacts with LUBAC as both a substrate in the LMP1 signaling pathway and as an inhibitor in the TLR signaling pathway, with opposite effects on NF-κB activation. How the TRAF1-LUBAC interaction results in distinct outcomes in different signaling complexes will require a precise understanding of the protein-protein interactions involved. Moreover, the fact that TRAF1 can be M1-ubiqutinated by LUBAC and phosphorylated by PKN1 suggest that post-translational modifications will be important in this regulation and need further study.

## Author Contributions

ME and TW wrote the manuscript with editorial input from AA-S. ME prepared the figures.

### Conflict of Interest Statement

The authors declare that the research was conducted in the absence of any commercial or financial relationships that could be construed as a potential conflict of interest.

## Abbreviations

C5: complement factor 5
cIAP: cellular inhibitors of apoptosis
EBV: Epstein Barr virus
ERK: extra-cellular signaling related kinase
HOIL, Heme oxidized IRP2 Ub ligase-1: a subunit of LUBAC
HOIP, HOIL interacting protein: a subunit of LUBAC
IκB: inhibitor of kB
IKK: inhibitor of κB kinase
K48-Ub: ubiquitin polymerized through the K48 position
K63-Ub: ubiquitin polymerized through the K63 position
LMP1: latent membrane protein 1 from EBV
LUBAC: linear ubiquitin assembly complex
MAPK: mitogen activated protein kinase
MATH: meprin and TRAF homology
M1-Ub: ubiquitin polymerized through the M1 position
mTOR: mammalian target of rapamycin
NEMO: NF-κB essential modulator (NEMO) also known as IKKγ
NIK: NF-κB inducing kinase
NLR: Nod-like receptor
NZF: Npl4 Zinc finger domains
RA: rheumatoid arthritis
RIPK1: receptor-interacting serine/threonine-protein kinase 1
SHARPIN, Shank-associated RN domain-interacting protein: a component of LUBAC
TAB: TAK binding protein
TAK: TGF β-activated kinase
TIR domain: Toll/IL-1 receptor domain
TLRs: toll-like receptors
TRADD, TNFR associated death domain;TRAFs: TNFR associated factors
TNFR: tumor necrosis factor receptor
TRIF: Tlr domain-containing adaptor inducing interferon-β.
